# The Designer Drug 3-Fluoromethcathinone Induces Oxidative Stress and Activates Autophagy in HT22 Neuronal Cells

**DOI:** 10.1007/s12640-018-9898-y

**Published:** 2018-04-14

**Authors:** Kamila Siedlecka-Kroplewska, Agata Wrońska, Grzegorz Stasiłojć, Zbigniew Kmieć

**Affiliations:** 1 Department of Histology, Medical University of Gdańsk, 1 Dębinki St, 80-211 Gdańsk, Poland; 2 Laboratory of Cell Biology, Department of Medical Biotechnology, Intercollegiate Faculty of Biotechnology UG-MUG, Medical University of Gdańsk, Gdańsk, Poland

**Keywords:** Designer drugs, Synthetic cathinones, 3-Fluoromethcathinone, Oxidative stress, Autophagy, Apoptosis

## Abstract

Synthetic cathinones are psychoactive substances, derivatives of a natural psychostimulant cathinone. Although many synthetic cathinones have lost their legal status in many countries, their abuse still continues worldwide. Recently, they have been reported to exert neurotoxic effects in vitro and in vivo. The molecular mechanisms of their action have not been fully elucidated. Recently, they have been linked to the induction of oxidative stress, autophagy, and apoptosis. The aim of this study was to investigate whether 3-fluoromethcathinone (3-FMC), a synthetic cathinone, is able to induce oxidative stress, autophagy, and apoptosis in HT22 immortalized mouse hippocampal cells. We found that treatment of HT22 cells with this compound results in a concentration-dependent increase in the intracellular production of reactive oxygen species. Moreover, 3-FMC induced concentration-dependent conversion of cytosolic LC3-I to membrane-bound LC3-II and formation of autophagic vacuoles. Additionally, the level of p62/SQSTM1 protein decreased after 3-FMC treatment, suggesting that accumulation of autophagic vacuoles resulted from activation rather than inhibition of autophagy. Our results also showed that 3-FMC at millimolar concentration is able to induce caspase-dependent apoptotic cell death in HT22 cells. Our findings suggest that abuse of 3-FMC may disturb neuronal homeostasis and impair functioning of the central nervous system.

## Introduction

Synthetic cathinones are psychoactive substances, derivatives of a naturally occurring alkaloid cathinone (Balint et al. 2009; Zawilska and Wojcieszak 2013). Recently, they have gained popularity as recreational drugs or “legal highs” (ACMD 2010a; EMCDDA 2017). The new psychoactive substances, also known as “designer drugs,” have emerged as legal alternatives to classic illegal drugs of abuse such as cocaine or 3,4-methylenedioxy-N-methyl-amphetamine (MDMA). Although many cathinones, e.g., 4-methylmethcathinone (mephedrone), 3,4-methylenedioxypyrovalerone (MDPV), 3,4-methylenedioxy-N-methylcathinone (methylone), 3-fluoromethcathinone (3-FMC), and 4-fluoromethcathinone (4-FMC), have lost their legal status in many countries, their abuse still continues worldwide (EMCDDA 2017; Assi et al. 2017).

The molecular mechanism of action of synthetic cathinones is based on their interaction with transporters of monoamine neurotransmitters such as dopamine, serotonin, and norepinephrine (Cozzi et al. 1999; Simmler et al. 2013, 2014; Baumann et al. 2013). Many synthetic cathinones such as mephedrone, methylone, and MDPV were shown to exhibit high blood-brain barrier permeability in an in vitro model (Simmler et al. 2013; Martínez-Clemente et al. 2013). Users compared effects of these substances with those elicited by MDMA and cocaine (Assi et al. 2017; Carhart-Harris et al. 2011). The “positive/desired” effects reported by users were euphoria, stimulation, and empathy (Winstock et al. 2011b; Assi et al. 2017; Carhart-Harris et al. 2011; Zawilska and Wojcieszak 2013; ACMD 2010b). However, it should be emphasized that adverse effects were also reported, regarding mostly the cardiovascular, nervous, and gastrointestinal systems. For instance, in the cardiovascular system, hypertension, tachycardia, chest pain, palpitations, cardiac arrest, and myocarditis were reported after the use of cathinones (Assi et al. 2017; Zawilska and Wojcieszak 2013; Winstock et al. 2011a; Prosser and Nelson 2012; ACMD 2010b). The toxic effects concerning the nervous system included hyperthermia, insomnia, headache, dizziness, memory problems, confusion, blurred vision, bruxism, paresthesias, mydriasis, tremors, seizures, hallucinations/delusions, anxiety, agitation, and psychosis (Vallersnes et al. 2016; Winstock et al. 2011a, b; Zawilska and Wojcieszak 2013; Prosser and Nelson 2012; ACMD 2010b). Symptoms regarding the gastrointestinal system were abdominal pain, nausea, vomiting, and anorexia (Zawilska and Wojcieszak 2013; Winstock et al. 2011a; Prosser and Nelson 2012; ACMD 2010b).

Many drugs of abuse have been demonstrated to impair cognitive skills and exert neurotoxic effects (den Hollander et al. 2012; Gardner et al. 2009; Parrott 2000; Reneman et al. 2001; Thompson et al. 2004). MDMA, an active component of “ecstasy,” was shown to be toxic to brain serotonin neurons (Reneman et al. 2001). There is evidence of hippocampal atrophy in chronic users of “ecstasy” (den Hollander et al. 2012; Gardner et al. 2009). Memory impairments in “ecstasy” users were also documented (Parrott 2000). Additionally, chronic methamphetamine abuse was shown to reduce hippocampal volume and hippocampal deficits correlated with impaired memory performance in its users (Thompson et al. 2004). Accumulating data suggest that synthetic cathinones may also be neurotoxic and impair functions of the nervous system (Hadlock et al. 2011; Marusich et al. 2012; López-Arnau et al. 2014, 2015; Martínez-Clemente et al. 2014; den Hollander et al. 2013). In mice, methylone induced astrogliosis in the hippocampus as well as dopaminergic and serotonergic impairment (López-Arnau et al. 2014). Its administration in rats caused the depletion of serotonin and its transporters’ levels (den Hollander et al. 2013). Noteworthy, mephedrone was reported to impair working memory in humans (Freeman et al. 2012). Interestingly, there are different results regarding its neurotoxicity in animal models (Hadlock et al. 2011; López-Arnau et al. 2015; Martínez-Clemente et al. 2014; Baumann et al. 2012; den Hollander et al. 2013, Angoa-Pérez et al. 2012, 2014). Some studies on long-term neurochemical effects of mephedrone in rodents suggest lack of neurotoxicity (Baumann et al. 2012; den Hollander et al. 2013; Angoa-Pérez et al. 2012, 2014), whereas other studies indicate that it exerts neurotoxic effects (Hadlock et al. 2011; Martínez-Clemente et al. 2014; López-Arnau et al. 2015). Probably, the discrepancies are due to different experimental design, e.g., species, dosage, and ambient temperature as well as relation of experimental conditions to drug pharmacokinetics and pharmacodynamics.

Recent in vitro studies have also shown that synthetic cathinones may exhibit neurotoxic properties. However, the precise molecular mechanisms of their action have not been fully elucidated. Noteworthy, mephedrone elicited cytotoxicity against cortical neurons isolated from mouse embryos (Martínez-Clemente et al. 2014). Pyrovalerone and its derivatives reduced the viability of human neuroblastoma SH-SY5Y cells (Wojcieszak et al. 2016). Our recent investigation revealed that 3-FMC inhibits growth and induces cell cycle arrest in HT22 immortalized mouse hippocampal cells (Siedlecka-Kroplewska et al. 2014). Recently, some synthetic cathinones have been demonstrated to induce oxidative stress, autophagy, and apoptosis in neuronal cells (Valente et al. 2017; Matsunaga et al. 2017). Methylone and MDPV induced oxidative stress, autophagy, and apoptosis in differentiated human neuroblastoma SH-SY5Y cells (Valente et al. 2017). Similarly, treatment of human neuroblastoma SK-N-SH cells with α-pyrrolidinononanophenone (α-PNP) led to oxidative stress, autophagy, and apoptotic cell death (Matsunaga et al. 2017). Taking into account these findings, the aim of this study was to examine whether 3-FMC induces oxidative stress, autophagy, and apoptosis in HT22 hippocampal cells. Our results provide evidence that the mechanism of action of this synthetic cathinone in HT22 cells involves induction of oxidative stress as well as activation of autophagy. We also found that 3-FMC at millimolar concentrations is able to induce caspase-dependent apoptotic cell death.

## Materials and Methods

### Chemicals

3-Fluoromethcathinone was purchased from LGC Standards (UK). Stock solutions of this compound were prepared in sterile physiological saline solution and diluted to indicated concentrations shortly before use. Rabbit anti-LC3 primary antibodies were purchased from Medical & Biological Laboratories Co. (Japan). Mouse anti-p62 antibody was obtained from Santa Cruz Biotechnology, Inc. (USA). Cy3-conjugated goat anti-rabbit secondary antibodies were obtained from Jackson ImmunoResearch Laboratories, Inc. (USA). Horseradish peroxidase-conjugated mouse anti-GAPDH primary antibodies, horseradish peroxidase-conjugated goat anti-rabbit secondary antibodies, and horseradish peroxidase-conjugated rabbit anti-mouse secondary antibodies were purchased from Sigma-Aldrich (USA). H_2_DCFDA (2′,7′-dichlorodihydrofluorescein diacetate) was obtained from Molecular Probes (USA). Hoechst 33342 was purchased from Sigma-Aldrich (USA). All other reagents, obtained from commercial suppliers, were of analytical grade.

### Cell Culture

The immortalized mouse hippocampal HT22 cell line was kindly provided by Professor M. Woźniak (Department of Medical Chemistry, Medical University of Gdańsk, Poland). Cells were maintained at 37 °C in a humidified atmosphere containing 5% CO_2_, in Dulbecco’s Modified Eagle’s Medium (Sigma-Aldrich, USA), supplemented with 10% heat-inactivated fetal bovine serum (Sigma-Aldrich, USA), 100 IU/ml penicillin (Sigma-Aldrich, USA), and 100 μg/ml streptomycin (Sigma-Aldrich, USA).

### Measurement of Intracellular Reactive Oxygen Species

HT22 cells were seeded in 6-well plates (2 × 10^5^cells per well) and allowed to attach for 24 h. Next, cells were incubated with 3-FMC for 45 or 90 min. Simultaneously, control cells were incubated in the absence of 3-FMC. Thirty minutes before the end of incubation with 3-FMC, H_2_DCFDA (final concentration 10 μM) was added. Cells were then washed, suspended in ice-cold phosphate-buffered saline (PBS), and analyzed for DCF fluorescence by flow cytometry (Becton Dickinson FACSCalibur, USA).

### Western Blotting Analysis

HT22 cells were incubated in the absence (control) or presence of 3-FMC for 24 h. After incubation, cell lysates were prepared using Mammalian Cell Extraction Kit (BioVision, Inc., USA). The total concentration of proteins in cell lysates was determined using the Bradford protein assay. Protein samples (45 μg of total protein per sample) were separated electrophoretically by SDS-PAGE (12%) and transferred onto PVDF membrane. The membrane was incubated with 5% non-fat dry milk in TBS (tris-buffered saline) at room temperature (RT) for 1 h. After washing with TBST (0.1% Tween20 in TBS), the membrane was incubated with specific rabbit anti-LC3 primary antibodies (1:4000) or mouse anti-p62 primary antibodies (1:200) at 4 °C overnight, and after subsequent washing incubated with appropriate horseradish peroxidase-conjugated secondary antibodies (1:10,000) for 2 h at RT. The membrane was also incubated with horseradish peroxidase-conjugated anti-GAPDH primary antibodies (1:50,000, 1 h at RT) for loading control. The bound antibodies were detected by the enhanced chemiluminescence method using the Chemiluminescent Peroxidase Substrate (Sigma-Aldrich, USA). The densitometric analysis of immunoreactive protein bands was performed using Quantity One Software (Bio-Rad, USA).

### Immunofluorescent Analysis

HT22 cells were seeded in 4-well tissue culture chamber (8 × 10^4^ cells per well) on PCA slide (Sarstedt, Germany) and allowed to attach for 24 h. Next, cells were incubated in the absence (control) or presence of 3-FMC for 24 h. Following incubation, cells were washed with PBS, fixed, and permeabilized for 5 min in cold methanol at − 20 °C. After washing with PBS, cells were incubated for 30 min (RT) with 10% FBS (fetal bovine serum, 10% FBS in PBS). Next, cells were washed with PBS and incubated with specific rabbit anti-LC3 primary antibodies (1:500) for 1 h (RT). After washing with PBS, cells were incubated with Cy3-conjugated anti-rabbit secondary antibodies (1:600) for 1 h (RT) in the dark. Following washing with PBS, cells were stained with 5 μg/ml Hoechst 33342 for 15 min (RT) in the dark. Next, samples were mounted in the Permafluor mounting medium (Thermo Scientific, USA) and covered with glass coverslips. Slides were examined by the confocal microscope system FV10i (Olympus, Japan). The images were obtained using × 60 objective lens.

### Hoechst 33342 Staining

Adherent cells undergoing cell death tend to detach from the surface of tissue culture flasks. In order to prevent cell loss and better examine nuclear morphology of 3-FMC-treated cells, we tried to improve our standard procedure of cell staining for confocal microscopy. HT22 cells were seeded in 6-well plates (2 × 10^5^ cells per well) and allowed to attach for 24 h. Next, cells were incubated in the absence (control) or presence of 3-FMC for 24 h. After incubation, all cells were collected, i.e., including cells detached from the surface of the culture flask as well as cells collected after trypsinization. Following washing with PBS, cells were cytocentrifuged (1 × 10^5^ cells per slide) onto microscopic poly-L-lysine-coated slides (Sigma-Aldrich, USA). After fixation and permeabilization in cold methanol for 5 min at − 20 °C, cells were washed with PBS and stained with 5 μg/ml Hoechst 33342 for 15 min (RT) in the dark. Next, samples were mounted in the Permafluor mounting medium (Thermo Scientific, USA) and covered with glass coverslips. Slides were examined by the confocal microscope system FV10i (Olympus, Japan). The images were obtained using × 60 objective lens.

### Annexin V-FITC/PI Assay

Phosphatidylserine externalization was examined using Annexin V-FITC Apoptosis Detection Kit (BD Pharmingen, USA). HT22 cells were seeded in 6-well plates (2 × 10^5^cells per well) and allowed to attach for 24 h. Next, cells were incubated in the absence (control) or presence of 3-FMC for 24 h. Following incubation, cells were stained with FITC-conjugated annexin V and PI according to the manufacturer’s protocol. Samples were analyzed by flow cytometry (Becton Dickinson FACSCalibur, USA).

### Caspase-3 Activity Assay

Caspase-3 activity was measured using FITC-conjugated Monoclonal Active Caspase-3 Antibody Apoptosis Kit I (BD Pharmingen, USA). HT22 cells were seeded in 6-well plates (2 × 10^5^ cells per well) and allowed to attach for 24 h. Next, cells were incubated in the absence (control) or presence of 3-FMC for 24 h. Following incubation, cells were stained with FITC-conjugated anti-active caspase-3 antibody according to the manufacturer’s protocol and flow cytometric analyses were performed (Becton Dickinson FACSCalibur, USA).

### Statistical Analysis

Statistical analysis was performed using Statistica 12 software (StatSoft, Poland). Data are expressed as means ± SD. Statistical differences between samples were evaluated using the non-parametric Mann-Whitney *U* test. Differences were considered significant at *p < 0.05 and **p < 0.01.

## Results

### Effect of 3-FMC on Generation of Reactive Oxygen Species

**Fig. 1:**
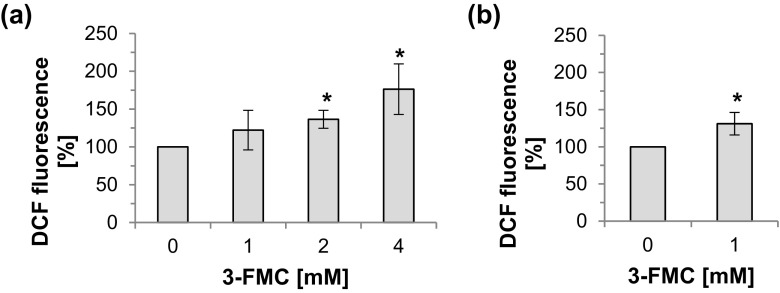
Effect of 3-FMC on intracellular ROS production in HT22 cells. HT22 cells were treated with 3-FMC for 45 min (**a**) or 90 min (**b**). Cells were analyzed by flow cytometry as described in Materials and Methods. Data are presented as means ± SD of three independent experiments, *n* = 4 (*n*, number of samples per each experimental point), *p < 0.05, statistically significant differences compared to control (untreated cells)

We have previously found that 3-FMC is cytotoxic to HT22 cells at relatively high, millimolar concentration since 24 h of treatment with 1, 2, or 4 mM 3-FMC reduced the viability of HT22 cells by 16, 34, and 76%, respectively (Siedlecka-Kroplewska et al. 2014). To find out whether the mechanism of action of 3-FMC involves oxidative stress, we examined the effect of this compound on the intracellular production of reactive oxygen species (ROS). Our results showed that the formation of ROS increased after treatment of HT22 cells with 3-FMC. Compared to control cells, exposure to 2 or 4 mM 3-FMC resulted in a statistically significant increase in ROS formation after 45 min (Fig. 1a), whereas 1 mM 3-FMC significantly induced ROS generation after 90 min of incubation (Fig. 1b).

### Detection of Autophagy in 3-FMC-Treated HT22 Cells

**Fig. 2:**
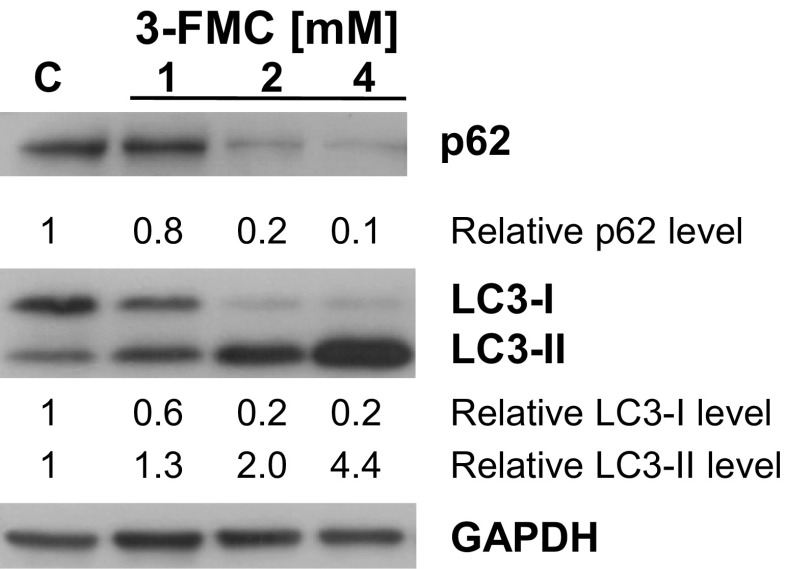
Detection of autophagy. HT22 cells were treated with 1, 2, or 4 mM 3-FMC for 24 h. The relative protein levels of LC3-I, LC3-II, and p62 normalized to loading control GAPDH were quantitated by densitometry as described in Materials and Methods. Similar results were obtained in three independent experiments. C—control, untreated cells

The microtubule-associated protein 1 light chain 3 (LC3) plays an important role in autophagy (Eskelinen 2005). During autophagy, the cytosolic form of LC3 (LC3-I) is conjugated with phosphatidylethanolamine forming the membrane-bound form of LC3 (LC3-II). Detection of LC3-II is a hallmark of the formation of autophagic vacuoles. To investigate the effects of 3-FMC on autophagic pathways, we examined the conversion of LC3-I to LC3-II. The western blotting analysis revealed that after 24 h of treatment of HT22 cells with 3-FMC, the level of LC3-II increased, indicating processing of LC3-I and formation of LC3-II. This effect was concentration-dependent and was most pronounced at the 3-FMC concentration of 4 mM (Fig. 2). The relative LC3-II level (normalized to loading control GAPDH) after exposure to 1, 2, and 4 mM 3-FMC was 1.3, 2.0, and 4.4, respectively. The relative LC3-I level after 3-FMC treatment decreased compared to control and for 1, 2, and 4 mM 3-FMC, it was equal to 0.6, 0.2, and 0.2, respectively (Fig. 2).

**Fig. 3:**
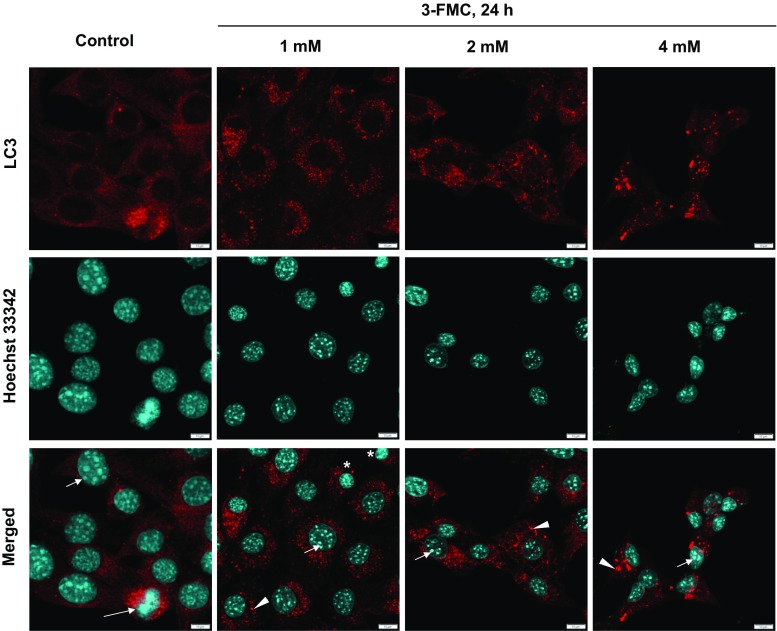
Immunofluorescent analysis. Confocal micrographs of HT22 cells treated with 1, 2, and 4 mM 3-FMC for 24 h. Cells were incubated with primary anti-LC3 antibodies. Following incubation with Cy3-conjugated secondary antibodies and Hoechst 33342, cells were examined by confocal microscopy as described in Materials and Methods. Data are representative of three independent experiments. Bars 10 μm, control—untreated cells, arrowheads—autophagic vacuoles, short arrows—nucleoli, long arrow—a cell undergoing mitosis, asterisks—newly formed cells after cell division

The immunofluorescent staining with anti-LC3 antibodies revealed the accumulation of LC3-positive dots in HT22 cells treated with 1, 2, or 4 mM 3-FMC for 24 h (Fig. 3), suggesting accumulation of autophagic vacuoles. It was particularly evident after exposure to 4 mM 3-FMC. In control cells, LC3 staining was mostly diffuse, indicative of cytosolic localization of LC3 protein (Fig. 3).

In order to find out whether the accumulation of autophagic vacuoles in HT22 cells results from activation or inhibition of autophagy, we evaluated the level of p62/SQSTM1 protein. The p62 protein, also known as sequestosome-1 (SQSTM1), interacts with ubiquitinated proteins targeting them for degradation by autophagy (Klionsky et al. 2012). Our results showed that its level in HT22 cells decreased after 3-FMC treatment (Fig. 2). The relative p62/SQSTM1 level (normalized to loading control GAPDH) after exposure to 1, 2, and 4 mM 3-FMC was 0.8, 0.2, and 0.1, respectively (Fig. 2).

### Detection of Cell Death

Our previous results revealed that treatment of HT22 cells with 3-FMC led to an increase in the number of cells in the sub-G_1_ fraction, indicative of apoptosis (Siedlecka-Kroplewska et al. 2014). In line with this finding, in the present study, we examined markers of apoptotic cell death such as phosphatidylserine externalization, caspase-3 activation, chromatin condensation, and fragmentation of cell nuclei.

**Fig. 4:**
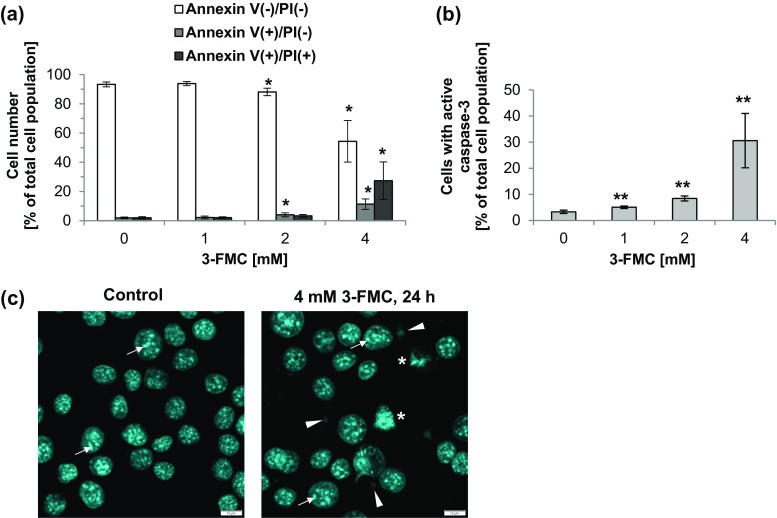
Detection of cell death. **a** Phosphatidylserine externalization induced by 3-FMC in HT22 cells (annexin V-FITC/PI staining, flow cytometry analysis). Cells were treated with 1, 2, or 4 mM 3-FMC for 24 h. Data are presented as means ± SD of three independent experiments. *n* = 4–5,*p < 0.05, statistically significant differences compared to control (untreated cells). **b** Caspase-3 activation in 3-FMC-treated HT22 cells (flow cytometry analysis). Cells were treated with 1, 2, or 4 mM 3-FMC for 24 h. Data are presented as means ± SD of three independent experiments. *n* = 4–7, **p < 0.01, statistically significant differences compared to control (untreated cells). **c** Confocal micrographs of HT22 cells treated with 4 mM 3-FMC for 24 h. Cells were stained with Hoechst 33342 as described in Materials and Methods. Data are representative of three independent experiments. Bars 10 μm, control—untreated cells, short arrows—nucleoli, arrowheads—fragments of cell nuclei, asterisks—cell nuclei with condensed chromatin

Loss of the plasma membrane asymmetry manifested by phosphatidylserine externalization belongs to early apoptotic events (Galluzzi et al. 2012). As shown in Fig. 4a, the effect of 3-FMC on phosphatidylserine externalization was concentration-dependent. The prominent changes were observed after treatment with 4 mM 3-FMC, when annexin V^+^/PI^−^ cells (corresponding to early apoptotic cells) and annexin V^+^/PI^+^ cells (corresponding to late apoptotic/necrotic cells) constituted about 11 and 27% of the total measured cell population, respectively (Fig. 4a). Exposure of HT22 cells for 24 h to 1 and 2 mM 3-FMC had a negligible effect on fractions of annexin V^+^/PI^−^ cells and annexin V^+^/PI^+^ cells.

We found that 3-FMC-induced cell death was associated with caspase-3 activation (Fig. 4b). The active caspase-3 participates in the executive stage of apoptosis (Galluzzi et al. 2012). After 24 h of incubation of HT22 cells with 1 or 2 mM 3-FMC, 5% and over 8% of the total measured cell population, respectively, showed caspase-3 activity (Fig. 4b). This effect was most prominent after 24 h of treatment with 4 mM 3-FMC, since the fraction of cells with active caspase-3 was then over 30% (Fig. 4b).

Changes of the nuclear morphology typical for apoptotic cell death include chromatin condensation and nuclear fragmentation (Prokhorova et al. 2015). The immunofluorescent analysis revealed that after exposure to 1 or 2 mM 3-FMC, the majority of HT22 cells exhibited intact cell nuclei with well visible nucleoli (Fig. 3). Noteworthy, at the same time, LC3-positive structures were present, indicative of autophagic vacuoles. Both accumulation of autophagic vacuoles and an intact nucleus are characteristics of cells undergoing autophagy (Liu and Ouyang 2014). Interestingly, after treatment with 1 mM 3-FMC, even cells undergoing mitosis were observed (Fig. 3). After 24-h exposure of HT22 cells to 4 mM 3-FMC, the majority of cells exhibited intact nuclear architecture, accompanied by the presence of autophagic vacuoles (Fig. 3). However, confocal micrographs revealed that the shape of cells treated with 4 mM 3-FMC changed and was more rounded in comparison to untreated cells and cells treated with lower concentrations of this drug, suggesting decreased cell adhesion to the surface of the culture flask. Annexin V/PI double staining analysis revealed a high number of dead cells after treatment with 4 mM 3-FMC (Fig. 4a). Considering that adherent cells undergoing death tend to detach from the surface of the culture flask and may be lost during washing steps, we modified our standard procedure of slides preparation for confocal microscopy as described in Materials and Methods. Noteworthy, the detached cells may be suspected of pronounced apoptotic nuclear alterations. Using our improved method, we detected the presence of nuclear fragmentation and chromatin condensation after 24 h of treatment with 4 mM 3-FMC (Fig. 4c).

## Discussion

Recently, several in vitro and in vivo studies have shown that synthetic cathinones may exert neurotoxic effects (López-Arnau et al. 2014; Siedlecka-Kroplewska et al. 2014, Matsunaga et al. 2017; Valente et al. 2017; Hadlock et al. 2011; Martínez-Clemente et al. 2014; López-Arnau et al. 2015; den Hollander et al. 2013; Marusich et al. 2012). There is evidence indicating that these drugs, especially at high concentrations, may cause neuronal cell death (López-Arnau et al. 2014; Siedlecka-Kroplewska et al. 2014; Matsunaga et al. 2017; Valente et al. 2017). The precise molecular mechanisms of their action have not been fully elucidated. In this study, we used HT22 immortalized mouse hippocampal cells as an in vitro model of neuronal cells. HT22 cell line is widely used to study glutamate toxicity as well as Alzheimer’s and Parkinson’s diseases (Kumari et al. 2012; Fukui et al. 2009; Yang et al. 2015; Jensen et al. 2017; Gliyazova and Ibeanu 2016; Kang et al. 2013). Noteworthy, the hippocampus is the unique region of the brain, where the neural stem cells can be found (Kempermann et al. 2015). It plays a crucial role in the formation of memory (Yonelinas 2013). Accumulating evidence suggests that drug abuse may lead to hippocampal atrophy and memory deficits (den Hollander et al. 2012; Gardner et al. 2009; Parrott 2000; Thompson et al. 2004).

In the present study, we demonstrate that 3-FMC induces oxidative stress in HT22 cells. After treatment with 3-FMC, we observed a concentration-dependent increase in the intracellular production of ROS. In agreement with our finding, recent in vitro studies have indicated that oxidative stress may also be involved in the mechanism of action of other synthetic cathinones such as α-PNP, methylone, and MDPV (Matsunaga et al. 2017; Valente et al. 2017). Exposure to α-PNP led to oxidative stress in human neuronal SK-N-SH cells (Matsunaga et al. 2017). Methylone and MDPV increased the production of reactive oxygen and nitrogen species in human dopaminergic SH-SY5Y cells (Valente et al. 2017). Interestingly, other psychostimulant drugs such as amphetamine or methamphetamine were also shown to induce oxidative stress in neuronal cells (Huang et al. 1997; Cadet and Brannock 1998; Brown and Yamamoto 2003; Tian et al. 2009; Huang et al. 2017). The designer drug N-benzylpiperazine (BZP) induced oxidative stress in human glioblastoma LN-18 cells (Persona et al. 2016). It is important to note that the overproduction of reactive oxygen or nitrogen species triggers oxidative damage of cellular structures and disturbs cellular homeostasis (Halliwell 1996).

We found that 3-FMC activates autophagy in HT22 cells. Noteworthy, the higher the 3-FMC concentration, the more pronounced were the markers of autophagy. Confocal microscopy revealed an increased number of LC3-positive structures in 3-FMC-treated HT22 cells. Moreover, we detected conversion of cytosolic LC3-I to membrane-bound LC3-II, indicative of the formation of autophagic vacuoles. The western blotting analysis revealed a concentration-dependent increase in LC3-II expression. The cytosolic LC3-I protein serves as a substrate to form the LC3-II protein, present in membranes of autophagosomes, nascent amphisomes, and nascent autolysosomes (Eskelinen 2005). Both accumulation of autophagic vacuoles and an elevated LC3-II level may suggest upregulation of autophagy; however, they may also indicate inhibition of autophagic flux associated with impaired degradation and reduced turnover of autophagosomes (Klionsky et al. 2012). Therefore, in order to better evaluate the autophagic status, we examined the level of p62/SQSTM1 protein. An increased level of p62/SQSTM1 was shown to correlate with the inhibition of autophagy, whereas its decreased level with activation of autophagy (Klionsky et al. 2012). We found that p62/SQSTM1 protein level decreased after 3-FMC treatment, suggesting that accumulation of autophagic vacuoles in HT22 cells resulted from activation rather than inhibition of autophagy.

Autophagy is an evolutionarily conserved process during which damaged or misfolded proteins as well as damaged cell organelles can be eliminated (Yang and Klionsky 2010). It is active at basal level in virtually all cells and serves mainly as a prosurvival mechanism underlying cellular homeostasis. In neuronal cells, it is essential to maintain their functions. Insufficient or impaired autophagic activity has been described in neurodegenerative disorders such as Alzheimer’s disease, Parkinson’s disease, amyotrophic lateral sclerosis, and HIV-associated neurocognitive disorders (Cai et al. 2016). However, autophagy may also function as a cell death mode known as autophagic cell death (Galluzzi et al. 2012). There is a limited number of studies demonstrating that cell death is executed by autophagy (Galluzzi et al. 2012). In most cases, autophagy appears to be a cytoprotective response activated by dying cells (Galluzzi et al. 2012; Kroemer and Levine 2008). Autophagy can be upregulated in response to nutrient depletion, hypoxia, or oxidative stress (Yang and Klionsky 2010). Many chemical compounds induce cellular stress and activate autophagy as an adaptive stress-response and a prosurvival mechanism (Yang et al. 2011; Eskelinen 2011). The cytoprotective role of autophagy may be related then to clearance of oxidized or aggregated proteins and damaged cell organelles. Accumulating evidence suggests that autophagy may be implicated in the mechanism of action of drugs of abuse. Numerous psychoactive substances including MDMA, methamphetamine, cocaine, α-PNP, methylone, and MDPV were found to induce autophagy (Li et al. 2014, 2016; Mercer et al. 2017; Kanthasamy et al. 2006; Chandramani Shivalingappa et al. 2012; Larsen et al. 2002; Cao et al. 2016; Matsunaga et al. 2017; Valente et al. 2017). Interestingly, methylone and MDPV were shown to induce both oxidative stress and autophagy in SH-SY5Y cells (Valente et al. 2017). α-PNP also led to oxidative stress induction as well as autophagy upregulation in SK-N-SH cells (Matsunaga et al. 2017). In the present study, oxidative stress induced by 3-FMC in HT22 cells may result in damage of proteins or organelles. Thus, upregulation of autophagy in 3-FMC-treated HT22 cells may appear as a consequence of disturbed cellular homeostasis. The decreased level of p62/SQSTM1 protein in 3-FMC-treated HT22 cells suggests autophagy activation with concomitant degradation of damaged proteins or damaged cellular organelles. Of note, p62 protein serves as a selective autophagy receptor involved in autophagic clearance of misfolded proteins, protein aggregates, or depolarized mitochondria, whose efficient elimination is critical for cellular homeostasis and survival (Rogov et al. 2014). p62 interacts with ubiquitinated autophagy substrates and LC3, becomes incorporated into autophagosomes, and subsequently degraded (Klionsky et al. 2012). Therefore, it can be speculated that autophagy activated in our experimental model appears as a prosurvival process. However, this hypothesis requires further investigation. Valente et al. demonstrated that antioxidants were able to attenuate generation of reactive oxygen and nitrogen species as well as partially inhibit autophagy and apoptosis induced by methylone and MDPV in SH-SY5Y cells, supporting the role of autophagy as a cellular self-defense response against oxidative stress (Valente et al. 2017).

Our results revealed that 3-FMC is able to induce apoptotic cell death in HT22 cells. This effect was prominent after treatment of cells with 4 mM 3-FMC; whereas at a lower concentration of this drug, it was negligible. The cell death mechanism was associated with caspase-3 activation and phosphatidylserine externalization. Morphological changes characteristic for apoptotic cell death such as chromatin condensation and fragmentation of cell nuclei were also observed. These findings corroborate results of our previous study (Siedlecka-Kroplewska et al. 2014). Four millimolar 3-FMC significantly increased the number of HT22 cells in the sub-G1 fraction (Siedlecka-Kroplewska et al. 2014), corresponding to the low molecular weight DNA fragments, indicative of apoptotic internucleosomal DNA fragmentation (Wlodkowic et al. 2011).

Taking into account the above findings, after treatment with 1 or 2 mM 3-FMC, only autophagy markers were observed in HT22 cells; whereas after exposure to 4 mM 3-FMC, both autophagic and apoptotic characteristics were detected. Thus, 3-FMC induced autophagy or both autophagy and apoptosis, depending on its concentration. In agreement with our results, recent in vitro studies also showed induction of both autophagy and apoptosis after treatment with other synthetic cathinones (Valente et al. 2017; Matsunaga et al. 2017). After treatment of HT22 cells with 1 or 2 mM 3-FMC, the number of dead cells was negligible, which supports the hypothesis that autophagy activation may be a cytoprotective cell response. However, toxicity of 4 mM 3-FMC was probably too high to be prevented by autophagy. Therefore, the number of dead cells dramatically increased. Induction of apoptosis by 4 mM 3-FMC in HT22 cells indicates that apoptotic pathways are involved in the mechanism of cell death. Numerous studies suggest that there is an interplay between autophagy and apoptosis (Thorburn 2008). Autophagic and apoptotic signaling pathways share some mediators, e.g., Beclin-1 interacts with anti-apoptotic Bcl-2 family proteins (Thorburn 2008). Autophagy as a prosurvival mechanism may block or delay apoptotic cell death. Intriguingly, caspase activation may serve as a molecular switch between autophagy and apoptosis (Wu et al. 2014). Activated caspases degrade autophagic proteins and inhibit autophagic response determining cell fate. In the present study, the number of HT22 cells with active caspase-3 significantly increased after treatment with 4 mM 3-FMC, suggesting a possible switch from autophagy to apoptosis. However, further studies are needed to confirm this hypothesis.

Our finding that relatively high, millimolar concentrations of 3-FMC exerted significant biological effects in neuronal HT22 cells is consistent with reports of other authors concerning in vitro studies on drugs of abuse. For example, methylone and MDPV reduced viability of SH-SY5Y cells by 60% at the concentration of 2.797 and 1.693 mM, respectively (Valente et al. 2017). Moreover, methylone affected viability of cultured cortical neurons and the calculated LD50 value after 24 and 48 h of incubation was over 1 mM (López-Arnau et al. 2014). Other drugs including MDMA or methamphetamine also exerted cytotoxic effects in vitro at millimolar concentrations (Li et al. 2014, 2016; Mercer et al. 2017; Huang et al. 2017). It is difficult to predict what would be the 3-FMC concentration in the human brain in vivo after its administration. It may depend on the administration route as well as the ability of this drug to penetrate the blood-brain barrier. The most common administration routes of synthetic cathinones reported by users are insufflation and oral ingestion (Freeman et al. 2012; Assi et al. 2017). The less common routes are intravenous, subcutaneous, intramuscular injections as well as rectal insertion, smoking, and insertion in the eye (eyeballing) (Assi et al. 2017). Assuming the average peripheral blood volume of 5 L and intravenous administration route, the calculated 3-FMC dose leading to its 1, 2, or 4 mM blood concentration is equal to 1.09, 2.18, and 4.35 g, respectively. Noteworthy, based on users’ self-reports, doses depend on the drug and the administration route and range from a few milligrams to over 1–2 g in a single session (Busardò et al. 2015). However, higher doses were also documented (Busardò et al. 2015; EMCDDA 2010). According to case reports concerning synthetic cathinone-related intoxications, users reported doses up to 7 g (EMCDDA 2010). Some users reported to use drugs over several consecutive days (Winstock et al. 2011b). The risk of overdosing seems to be high, since users experience a desire to redose (Winstock et al. 2011b; Freeman et al. 2012). It should be emphasized that there are numerous reports on acute and lethal intoxication with synthetic cathinones (Boulanger-Gobeil et al. 2012; Winder et al. 2013; Corkery et al. 2012; Adamowicz et al. 2013; Marinetti and Antonides 2013; Cosbey et al. 2013; Antonowicz et al. 2011; Lusthof et al. 2011; Wood et al. 2011; Murray et al. 2012; Brandt et al. 2010; Schifano et al. 2012; Maskell et al. 2011; Warrick et al. 2012; Wikström et al. 2010; Imam et al. 2013).

In conclusion, our results provide evidence that 3-FMC induces oxidative stress and activates autophagy in HT22 neuronal cells. We propose that autophagy triggered by oxidative damage in 3-FMC-treated HT22 cells may appear as a cellular defense mechanism, however, when it cannot prevent toxicity of a high dose of 3-FMC apoptotic pathways become activated. Further studies may help understand molecular mechanisms of 3-FMC neurotoxicity. Noteworthy, the implication of oxidative stress in the mechanism of action of 3-FMC strongly suggests that abuse of this synthetic cathinone may disturb neuronal homeostasis and impair functioning of the central nervous system.
